# Supplementary material for: The changing meaning of ‘home’ in the work of South African women academics during the pandemic-enforced lockdown
Source: PLoS One. 2023 Jan 25;18(1):e0280179. doi: 10.1371/journal.pone.0280179 (PMC9876272; doi:10.1371/journal.pone.0280179)
Supplement: Attachment — Submitted filename: Response to Reviewers .docx [file pone.0280179.s001.docx]

**Response to Reviewers**

**PONE-D-22-15619**

The changing meaning of home in the work of South African women academics during the pandemic-enforced lockdown

| **Reviewer #1** | **Author’s response** |
| --- | --- |
| I think the authors should emphasise the problem that the concept of "home" has changed for women academics. They mention that the meaning of home has changed from being a place of refuge to a place congested, competitive and constrained. However, they do not sufficiently highlight the implications of women not having a refuge or the complications and threats women academics face for not matching their male counterparts' performance. | The authors contend that the implications have been discussed at length under the revised *Impact* and *Discussion* sections.  In addition, the following paragraph has been added:  Examining the implications for altered spaces are crucial for ensuring women's academic output is not disproportionately affected by the enforced pandemic lockdown; and in that way safeguarding the career trajectories of female academics. The gendered nature of childcare and domestic tasks as well as single-parent households, the majority of which are female-headed, have drastically affected female academics and their futures in the academy. |
| I am also wondering about the relevance of the research for the future. What do these results imply for women academics in the future, and how can policies be implemented to support women academics who have to perform and prove themselves? | The following has been included to address research for the future:  Future theoretical work will have to account for the micropolitics of the changing meanings of home and how exactly space is claimed, conceded, and constantly negotiated between academic women and their partners, as well as the children and other occupants under the same roof. In this way, under work from home policies, academic institutions are no longer interfacing with their employees alone, but all members of the employees’ household. Models for how these relationships are enacted and managed must be conceptualised. Thus, clear policy directions for higher education institutions to remedy the negative consequences of the pandemic lockdown for women's academic work at home and their professional futures need to seriously be taken into account. There is no doubt that science and innovation benefit from diversity. We need inclusive universities. To this end, the implications that the loss of women’s scientific expertise to the academy needs to be seriously considered. |
| **Specific comments:** Data: the authors should add descriptive statistics related to the sample demographics. They mention that the sample includes different levels of academia, but they do not provide frequencies (thus, how many from the sample were from which level). They also do not indicate the age, race, household size or institution distributions. Therefore they keep the reader wondering who the respondents were and if the sample is representative of female South African academics. There is most likely a selection bias in that female academics that were active and managed to adapt to the "new normal" did not take part in the survey as they did not have the time. | There is already an [article](https://doi.org/10.1016/j.respol.2021.104403) published that utilizes descriptive statistics from this particular dataset. Thus, the purpose of this article is to focus on the notion of “home”.  Demographic data such as age, race and institution distribution were not permitted to be asked in the survey according to the ethical permissions granted. |
| Furthermore, it seems that the data is not available to the readers. Thus, the reader cannot answer questions by exploring the data. I believe adding demographic variables can result in a more in-depth analysis. From an "intervention" point of view, this is especially important to determine which female academics suffered the most. | The research ethical permissions granted by Stellenbosch university – under the conditions that data will be anonymized; as well as that no demographic data will be shared to ensure participants privacy. We realize that demographic data such as race, age would be beneficial however, we could only ascertain career stage and no of children when it came to any demographic data. |
| Interestingly, in the narrative, I see no mention of the academia missing the interaction and conversation with their peers. Academics travel to conferences and workshops, share their research and ideas, and collaborate on various research projects. I believe that not having these interactions also negatively affected female researchers' mental states and productive capabilities. | It is important to address the notion that several participants lamented about missing interactions with their peers, as one academic and early-career mother of 2 children in primary school stated: “I miss my colleagues. I miss having a laugh in the passage. I miss being real with people. This remote meeting system only allows so much body language and I know that there are people who are suffering, and we are all blissfully unaware”. Similarly, a mid-career academic with no children uttered a similar tone of frustration: “I am already missing my colleagues - chatting, banging hands and smelling a bright new day outside the gate. I miss my workplace. We take those informal greetings on the bus for granted. Lord hear us. We want to go back to the old normal. The new normal is not good for emotional balance”. A mid-career academic without children lamented: “I am eagerly awaiting my return to my campus office. I love the academic, emotional freedom my office affords me. I miss the social interactions, the joy and comradeship at a colleague's elation, a manuscript is accepted for publication”. These reflections were not random but pertinent in our dataset.  We know that travel to conferences and workshops often include sharing research and ideas, and time and again, this leads to collaboration on various research projects. It is evident that not having these interactions negatively affected female researchers' mental states and productive capabilities which one mid-career mother of 2 pre-schoolers and a child in primary school affirmed: “Also because of the lockdown, I should have travelled to 4 international conferences, which would have given me proceedings, but that was cancelled. So my research output for this year is going to be close to zero”. |

Sincerely,

The Authors (31 October 2022)
